# Adipose stem cells from chronic pancreatitis patients improve mouse and human islet survival and function

**DOI:** 10.1186/s13287-017-0627-x

**Published:** 2017-08-30

**Authors:** Lili Song, Zhen Sun, Do-sung Kim, Wenyu Gou, Charlie Strange, Huansheng Dong, Wanxing Cui, Gary Gilkeson, Katherine A. Morgan, David B. Adams, Hongjun Wang

**Affiliations:** 1 Department of Surgery, Medical University of South Carolina, BSB 641, 173 Ashley Avenue, Charleston, SC 29425 USA; 2 Department of Medicine, Medical University of South Carolina, Charleston, SC USA; 3 Medstar Georgetown University Hospital, Washington, DC USA; 4 Department of Regenerative Medicine and Cell Biology, Medical University of South Carolina, Charleston, SC USA

**Keywords:** Adipose stem cells, Chronic pancreatitis, Islet transplantation, Islet survival

## Abstract

**Background:**

Chronic pancreatitis has surgical options including total pancreatectomy to control pain. To avoid surgical diabetes, the explanted pancreas can have islets harvested and transplanted. Immediately following total pancreatectomy with islet autotransplantation (TP-IAT), many islet cells die due to isolation and transplantation stresses. The percentage of patients remaining insulin free after TP-IAT is therefore low. We determined whether cotransplantation of adipose-derived mesenchymal stem cells (ASCs) from chronic pancreatitis patients (CP-ASCs) would protect islets after transplantation.

**Methods:**

In a marginal mass islet transplantation model, islets from C57BL/6 mice were cotransplanted with CP-ASCs into syngeneic streptozotocin-treated diabetic mice. Treatment response was defined by the percentage of recipients reaching normoglycemia, and by the area under the curve for glucose and c-peptide in a glucose tolerance test. Macrophage infiltration, β-cell apoptosis, and islet graft vasculature were measured in transplanted islet grafts by immunohistochemistry. mRNA expression profiling of 84 apoptosis-related genes in islet grafts transplanted alone or with CP-ASCs was measured by the RT^2^ Profiler™ Apoptosis PCR Array. The impact of insulin-like growth factor-1 (IGF-1) on islet apoptosis was determined in islets stimulated with cytokines (IL-1β and IFN-γ) in the presence and absence of CP-ASC conditioned medium.

**Results:**

CP-ASC-treated mice were more often normoglycemic compared to mice receiving islets alone. ASC cotransplantation reduced macrophage infiltration, β-cell death, suppressed expression of TNF-α and Bcl-2 modifying factor (BMF), and upregulated expressions of IGF-1 and TNF Receptor Superfamily Member 11b (TNFRSF11B) in islet grafts. Islets cultured in conditioned medium from CP-ASCs showed reduced cell death. This protective effect was diminished when IGF-1 was blocked in the conditioned medium by the anti-IGF-1 antibody.

**Conclusion:**

Cotransplantation of islets with ASCs from the adipose of chronic pancreatitis patients improved islet survival and islet function after transplantation. The effects are in part mediated by paracrine secretion of IGF-1, suppression of inflammation, and promotion of angiogenesis. ASCs from chronic pancreatitis patients have the potential to be used as a synergistic therapy to enhance the efficacy of islet transplantation following pancreatectomy.

## Background

Chronic pancreatitis is a disease characterized by chronic inflammation, fibrosis, and scaring in the pancreas parenchyma, followed by irreversible destruction of exocrine tissue [1]. In patients with long-term chronic pancreatitis, loss of pancreatic islet cells and diabetes can occur when severe fibrosis develops at the late stage of the disease progression. Total pancreatectomy with islet autotransplantation (TP-IAT) has been shown to be an effective treatment option for well-selected patients to prevent maladaptive intractable pain and to avoid pancreatogenic diabetes. However, up to 50% of islets undergo apoptosis caused by stresses induced during islet harvesting and transplantation [2, 3], and therefore less than one-third of TP-IAT patients remain diabetes free after the treatment, although the majority of patients were diabetes free before total pancreatectomy [4]. Instant blood-mediated inflammatory reaction, proinflammatory cytokines, and hypoxia are some of the many factors that contribute to β-cell death [5]. Another stress on transplanted islets occurs when the rich vasculature of pancreatic islets is disrupted by collagenase digestion during harvest, exposing islets to hypoxia and nutrient deprivation until the revascularization process is completed 10–14 days later [3, 6–8]. As the quality of islets harvested from patients suffering from chronic pancreatitis is usually poor because of chronic inflammation within the pancreas, strategies that promote angiogenesis and facilitate islet engraftment after transplantation would likely improve outcomes of islet autotransplantation, and would be of benefit in the more complex environment of allogeneic or xenogeneic islet transplantation [9].

Mesenchymal stem cells (MSCs) are multipotent adult stem cells that can be isolated from tissues of mesodermal origin including bone marrow, adipose tissue, and umbilical cord. MSC therapy has emerged as a novel biopharmaceutical approach because of the cells’ broad spectrum of immune-modulatory actions affecting both innate and adaptive immune systems, and their tissue-protective effects [10]. Injection of MSCs prolongs survival of skin, kidney, and cardiac allografts in allogeneic transplantation settings by inhibiting T-cell proliferation and inducing donor-specific allograft tolerance [11–15]. In rodent models, cotransplantation of syngeneic MSCs derived from multiple sources improved islet graft function in diabetic recipients [16–21]. Importantly, MSCs can migrate to sites of injury including pancreatic islets after intravenous injection [22, 23]. They can provide trophic support in local tissues, and can promote β-cell engraftment and regeneration [24]. MSCs exert their protective function for islets via downregulation of TNF-α and IFN-γ, and by increasing production of IL-10, hepatocyte growth factor (HGF), TGF-β, vascular endothelial growth factor (VEGF), and indoleamine 2,3-dioxygenase [25]. Adipose-derived mesenchymal stem/stromal cells (ASCs), isolated from fat tissue after liposuction, can be expanded easily in culture in large numbers, and have become an attractive alternative source of stem cells for therapy. Compared to MSCs from other tissues, ASCs have several advantages including ready accessibility, broader multipotent differentiation ability, better immunoregulatory effects, and greater secretion of angiogenic factors [26, 27].

There is evidence from small clinical trials that cotransplantation of bone marrow stem cells together with allogeneic islets provides improved glycemic control in patients with type 1 diabetes [28]. Cotransplantation of MSCs and islets as a synergistic therapy for islet auto-transplantation has potential clinical significance. No significant differences in phenotypes, differentiation abilities, or growth factor secretions were observed in MSCs harvested from healthy donors and CP patients (Wang, unpublished data). Whether patient-derived ASCs, especially those isolated from chronic pancreatitis patients (CP-ASCs), can protect islets from cell death after transplantation has yet to be evaluated. In this study, ASCs isolated from adipose tissue of chronic pancreatitis patients were used in coculture/transplant with mouse or human islets; islet death and function were compared in ex-vivo cell culture and in vivo after transplantation. Our data indicate that CP-ASCs enhance survival and function of islets, at least in part, through paracrine secretion of insulin-like growth factor-1 (IGF-1) and suppression of inflammation. Our study offers evidence for the potential mechanisms of how cotransplantation of CP-ASCs can protect islets from death after transplantation. Such a therapeutic approach can be readily translated to enhance efficacy of autologous islet transplantation for chronic pancreatitis patients, and can serve as a strong platform for allogeneic and xenogeneic islet transplantations.

## Methods

### Mice

Male C57BL/6 and NOD-SCID mice at 6–8 weeks of age (Jackson Laboratory, Bar Harbor, ME, USA) were used as donors or recipients in this study. All mouse surgical procedures were approved by the Animal Care Committee at the Medical University of South Carolina (protocol #AR3055).

### Patient ASC isolation and culture

Abdominal adipose tissues were obtained from chronic pancreatitis patients undergoing pancreatic surgery. All patients gave informed consent for the study under protocols approved by the Medical University of South Carolina Internal Review Board (Pro00028011). Adipose tissue was washed with PBS and then digested in 0.1% collagenase type II (Sigma Aldrich, St. Louis, MO, USA) for 20 min. Digestion was stopped by adding an equal amount of complete medium for ASCs (DMEM/F12, 1:1, supplemented with 10% FBS, 1% penicillin, and streptomycin). The cell mixture was filtered through a 100-μM mesh to remove debris. Cells were spun down, resuspended in complete medium at a concentration of 1 × 10^5^ cells/ml, and cultured in a 5% CO_2_ atmosphere at 37 °C. Floating cells were removed 8 hours after initial seeding. Cells were split when the monolayer reached 90% confluence, and were used between passages 3 and 6.

### Flow cytometric analysis of cellular markers of CP-ASCs

CP-ASCs were harvested and washed with PBS containing 5 mmol/L EDTA. After centrifugation, cells were resuspended in FACS buffer (PBS containing 250 μg/ml RNase A), and incubated with anti-CD29, anti-CD73, anti-CD44, anti-CD166, or anti-CD31 antibodies conjugated with fluorescent dyes or their corresponding isotype controls for 30 min in the dark at room temperature. Expression of cellular markers was analyzed on a BD FACSAria II flow cytometer (Becton Dickinson, Franklin Lakes, NJ, USA). Percentages of positive cells were calculated.

### Differentiation ability of CP-ASCs

ASCs were differentiated into adipocytes, chondroblasts, or osteoblasts using cell differentiation kits from Qiagen according to the manufacturer’s recommendations. Presence of adipocytes was identified by oil red staining; chondrocytes were identified by Alcian blue staining; and osteoblasts were identified by alkaline phosphatase staining as described previously [29].

### Islet preparation, diabetes induction, and islet transplantation

Islets were isolated from C57BL/6 mice by collagenase digestion as described previously [30]. Human islets from cadaveric donors were harvested at the Georgetown University. C57BL/6 or NOD-SCID mice were rendered diabetic by streptozotocin (STZ) injection (140 mg/kg, i.p.). At 5–7 days post injection, mice with blood glucose > 350 mg/dl were used as recipients. For each transplant, 125–150 hand-picked islets cocultured with CP-ASCs for 24 hours were transplanted together with 1 × 10^4^ CP-ASCs under the kidney capsule of the recipient. Control mice received only islets cultured in normal medium. Nonfasting blood glucose levels were measured twice per week for 60 days using an ACCU-CHEK glucometer (LifeScan, Mountain View, CA, USA). Mice with blood glucose levels < 200 mg/dl were considered normoglycemic.

### Immunohistochemistry staining

Islet grafts, together with part of the kidney, were collected, embedded in OCT, and snap frozen in liquid nitrogen. Tissues sections were stained with guinea pig anti-insulin polyclonal antibody (for β cells), or with anti-F4/80 antibody (for infiltrated macrophages) (both from Sigma-Aldrich). Phycoerythrin (PE) or FITC-conjugated anti-guinea pig, or PE-anti-rabbit secondary antibodies were used to detect expression of insulin or macrophages in transplanted islets.

### Terminal deoxynucleotidyl transferase-mediated dUTP nick end-labeling (TUNEL) quantification of apoptosis

Apoptotic cells in transplanted islet grafts were identified in OCT-embedded tissue sections using a Fluorescein In Situ Cell Death Detection Kit (Roche Diagnostics) based on the manufacturer’s instructions.

### Intravenous glucose tolerance test

Mice were fasted overnight (16 hours), and then injected with 1 mg/g body weight of glucose (i.v.). Blood samples were taken from the tail vein before (0) and 15, 30, 60, 90, and 120 min after glucose injection. Blood glucose levels were determined by glucometer and the area under the curve was calculated using standard methods. Blood samples were also collected from the tail vein at 0, 15, and 30 min after glucose injection at 7 and 14 days post transplantation. Serum was separated from whole blood by centrifugation, and c-peptide levels were measured using the C-peptide ELISA kit (ALPCO) according to the manufacturer’s recommendation.

### RT^2^ profiler™ PCR array for apoptosis in human islet grafts

At 3 days post islet transplantation, recipients were sacrificed by overdose of ketamine and xylazine. The graft-bearing kidney was exposed. Islet grafts that can be easily identified by their white color under a thin layer of kidney capsule were separated from the kidney using sharp forceps, and immediately immersed into the RNA lysis buffer provided by the RNeasy mini kit (QIAGEN). RNA was then extracted according to the manufacturer’s instructions. Following cDNA synthesis, expression of 84 apoptosis-related genes was analyzed using the RT^2^ Profiler™ PCR Array Human Apoptosis kit (Qiagen) using the CFX96 Touch™ Real-Time PCR Detection System from Biorad (Hercules, CA, USA).

### Quantitative real-time (RT)-PCR analysis

Quantitative real-time PCR was performed using a Bio-Rad CFX96 detection system with the SYBR Green Supermix kit. The following primers pairs were used for RT-PCR analysis: BMF, forward primer 5′-ATGGAGCCATCTCAGTGTGTG-3′ and reverse primer 5′-CCCCGTTCCTGTTCTCTTCT-3′; TNF, forward primer 5′-GGAGAAGGGTGACCGACTCA-3′ and reverse primer 5′-CTGCCCAGACTCGGCAA-3′; TNFRSF11B, forward primer 5′-GGCAACACAGCTCACAAGAA-3′ and reverse primer 5′-CGCTGTTTTCACAGAGGTCA-3′; IGF-1, forward primer 5′-TGGATGCTCTTCAGTTCGTG-3′ and reverse primer 5′-TGGTAGATGGGGGCTGATAC-3′; and ACTIN, forward primer 5′-TCACCCACACTGTGCCCATCTACG-3′ and reverse primer 5′-CAGCGGAACCGCTCATTGCCAATG-3′.

### Measurement of IGF-1 concentration in medium

Mouse islets were cultured in serum-free medium in the presence or absence of CP-ASCs. Cell culture supernatants were collected 24, 48, or 72 hours later from islets cultured alone, islets cocultured with CP-ASCs, or CP-ASCs cultured alone. Concentrations of mouse and human IGF-1 in supernatants were measured using ELISA kits for mouse or human IGF-1 (RayBiotech) according to the manufacturer’s recommendation.

### Conditioned medium

CP-ASCs were grown in complete medium until reaching 90% confluence, at which point the medium was replaced with serum-free medium containing only antibiotics. The conditioned medium was collected 48 hours later, centrifuged at 500 × *g* for 3 min, filtered through a 0.22-μm filter, snap frozen, and stored at –80 °C for future use.

### Measurement of cytokine-induced apoptosis

Mouse islets cultured in normal or conditioned medium were stimulated with IL-1β (100 U/ml) and IFN-γ (1000 U/ml) for 24 hours in the presence or absence of the anti-human IGF-1 antibody (AF-291-NA; R&D Systems). Cell death was measured by colorimetric assay in medium using a lactate dehydrogenase (LDH) cytotoxicity detection kit (Clontech, Mountain View, CA, USA), and in cell lysates using a Cell Death Detection ELISA Kit that detects histone-associated DNA fragments in mononucleosomes and oligonucleosomes (Roche).

### Statistical analysis

The percentage of mice reaching normoglycemia was plotted using Kaplan–Meier software, and differences in graft survival were compared by log-rank test. Data are expressed as mean ± SEM. Differences between groups were compared for statistical significance by ANOVA or Student’s *t* test for multiple comparisons if needed; *p* < 0.05 denoted significance.

## Results

### Characterization of CP-ASCs

**Fig. 1:**
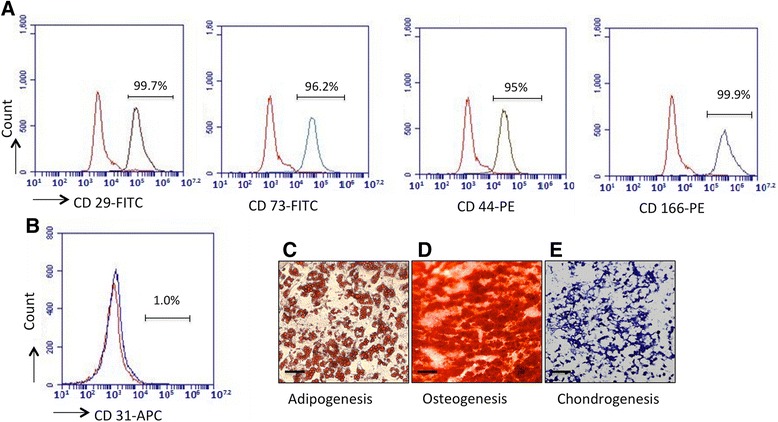
Characterization of CP-ASCs. Expression of (**a**) CD29, CD73, CD44, and CD166 and (**b**) CD31 in CP-ASCs measured by flow cytometry. *Red lines*, cells stained with corresponding isotype controls; *gray lines*, cells stained with individual antibody. Micrographs of (**c**) CP-ASC-derived adipocytes (oil red staining), (**d**) osteocytes (Alizarin red staining), and (**e**) chondrocytes (Toluidine blue staining). *Scale bar* =100 μm. *PE* phycoerythrin (Color figure online). *FITC* Fluorescein isothiocyanate, *APC* Allophycocyanin

We first characterized ASCs harvested from CP patients. CP-ASCs were fibroblast-like and exhibited adherence to plastic cell culture plates. They were positive for CD29, CD73, CD44, and CD166 (Fig. 1a), and were negative for CD31 (Fig. 1b). Their multipotency was confirmed by their ability to differentiate into adipocytes, osteocytes, and chondrocytes under specific induction conditions (Fig. 1c–e).

### Mouse islets cotransplanted with CP-ASCs showed better survival and function after syngeneic islet transplantation

**Fig. 2:**
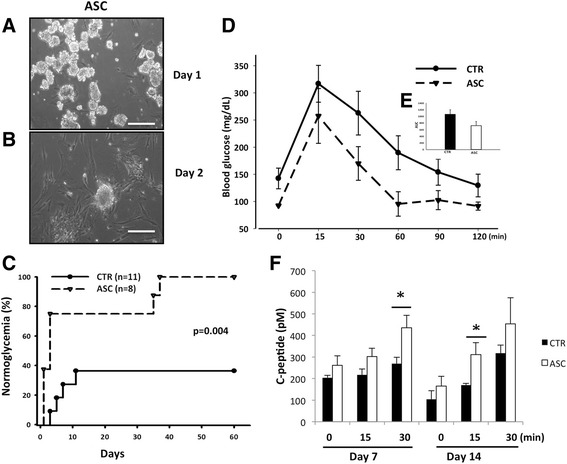
Survival of mouse islets cotransplanted with CP-ASCs in a syngeneic islet transplantation model. Micrographs of islets at 1 day (**a**) and 2 days (**b**) after coculture with CP-ASCs. *Scale bar* = 50 μm. **c** Percentage of C57BL/6 recipients achieving normoglycemia after receiving islets alone (*CTR*) or islets with CP-ASCs (*p* = 0.004 vs CTR, log-rank test). **d** Blood glucose levels of CTR and CP-ASCs mice during the IVGTT. **e** Area under the curve (AUC) of the IVGTT in CTR and ASC mice. **f** Serum c-peptide levels at 7 and 14 days post transplantation. *p < 0.05, ANOVA test. *ASC* mice receiving islets together with adipose-derived mesenchymal stem cells from chronic pancreatitis patients, *CTR* mice receiving islets alone

We next determined whether cotransplantation with CP-ASCs enhances islet survival and function post transplantation using a C57BL/6 syngeneic islet transplantation model. Islets from C57BL/6 mice were first cultured with CP-ASCs (Fig. 2a, b), and then cotransplanted with 1 × 10^4^ CP-ASCs into streptozotocin (STZ)-induced diabetic recipients. Islets cultured alone and transplanted without the addition of CP-ASCs were used as controls. When 125–150 control islets were transplanted into syngeneic recipients, 4/11 (36%) recipients reached normoglycemia by the end of the experiment (Fig. 2c). In contrast, 100% of mice that received islets and CP-ASCs (*n* = 8) reached normoglycemia by 37 days post transplantation, and remained normoglycemic until the end of the experiment (60 days post transplantation, Fig. 2c). To determine whether there was a difference in islet function between control and CP-ASC islet grafts, an intravenous glucose tolerance test (IVGTT) was performed in recipients that reached normoglycemia. Glucose levels were lower in mice receiving ASCs and islets than in controls at all times after glucose challenge (Fig. 2d), and the area under the curve was reduced (Fig. 2e). Moreover, we measured serum c-peptide levels as indications of islet function after glucose stimulation at 7 and 14 days post transplantation. Our data confirmed that islets cotransplanted with CP-ASCs had higher c-peptide levels compared to CTR islets at both time points measured (Fig. 2f), suggesting improved islet function in islets cotransplanted with CP-ASCs.

### Reduced cell death and macrophage infiltration in mouse islet grafts cotransplanted with CP-ASCs

**Fig. 3:**
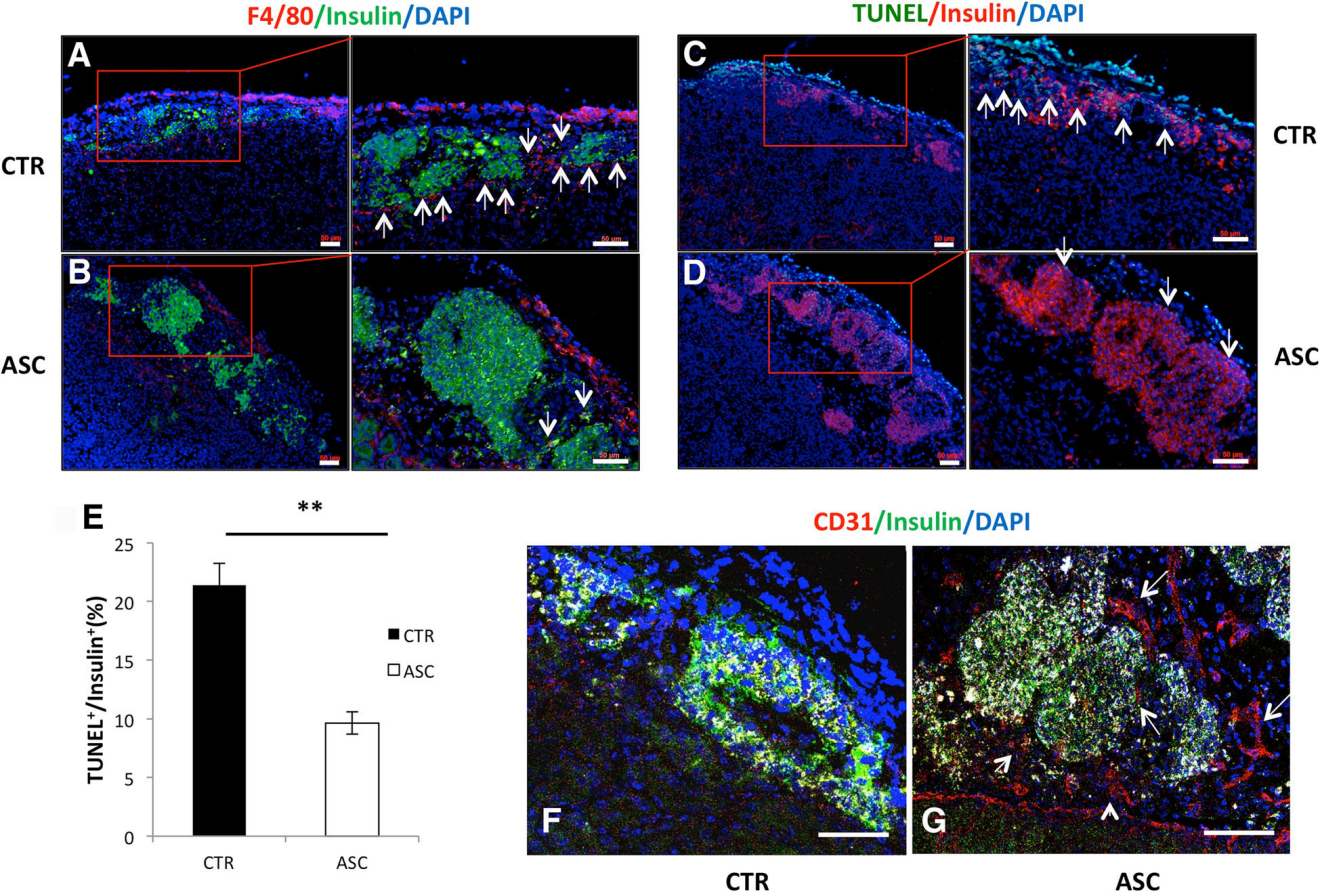
Immunohistochemical analysis of mouse islet grafts. **a**, **b** Analysis 3 days post transplant shows more macrophages and less insulin in control islets (*CTR*, **a**) compared to islets cotransplanted with CP-ASCs (*ASC*, **b**). *Red*, F4/80^+^ cells; *green*, insulin. *Arrows* point to macrophages. **c**, **d** More cell death observed in control (**c**) compared to CP-ASC islets (**d**) identified by TUNEL assay. *Green*, apoptotic cells; *red*, β cells. *Arrows* point to TUNEL^+^insulin^+^ cells. **e** Quantification of TUNEL^+^ among insulin ^+ ^cells in control or CP-ASC cotransplanted islets. **p < 0.05, Student’s *t* test. **f**, **g** Tissues 10 days post transplant. Immunohistochemical staining of endothelial cells (CD31^+^) is less in control islets (**f**) compared to ASC islet grafts (**g**) using the anti-CD31 antibody. *Red*, CD31^+^ cells; *green*, insulin^+^ cells. *Arrows* point to CD31^+^ cells. Tissue sections from at least three individual mice for each condition were analyzed. **a**–**e** Observed using the ZEISS AxioImager M2 Imaging System. **f**, **g** Observed using a Leica SP5 confocal microscope. *Scale bar* = 50 μm. *ASC* adipose-derived mesenchymal stem cell, *TUNEL* terminal deoxynucleotidyl transferase-mediated dUTP nick end-labeling (Color figure online)

Macrophage infiltration and nonimmune-related inflammation contribute to early islet death after transplantation. To understand the mechanisms by which CP-ASC cotransplantation was protective, we assessed infiltration of macrophages, islet death, and insulin expression in mouse islet grafts 3 days after transplantation by immunohistochemistry. Islets cotransplanted with CP-ASCs exhibited dramatically reduced macrophage infiltration (Fig. 3a, b) and cell death as measured by immunohistochemical staining (Fig. 3c, d) and quantification (Fig. 3e). Consistent with the cell death data, ASC islets showed stronger insulin staining.

Delayed islet graft revascularization can also contribute to graft death. We measured islet revascularization by immunohistochemical staining of endothelial cells using an anti-CD31 antibody in islet grafts 10 days post transplantation. Islets cotransplanted with CP-ASCs showed a significant increase in the amount vessel-like structure made of CD31^+^ cells around the islet grafts compared to control grafts (Fig. 3f, g), suggesting improved revascularization in ASC islet grafts.

### Cotransplantation with CP-ASCs modified gene expression profiles in human islet grafts transplanted into NOD-SCID recipients

**Fig. 4:**
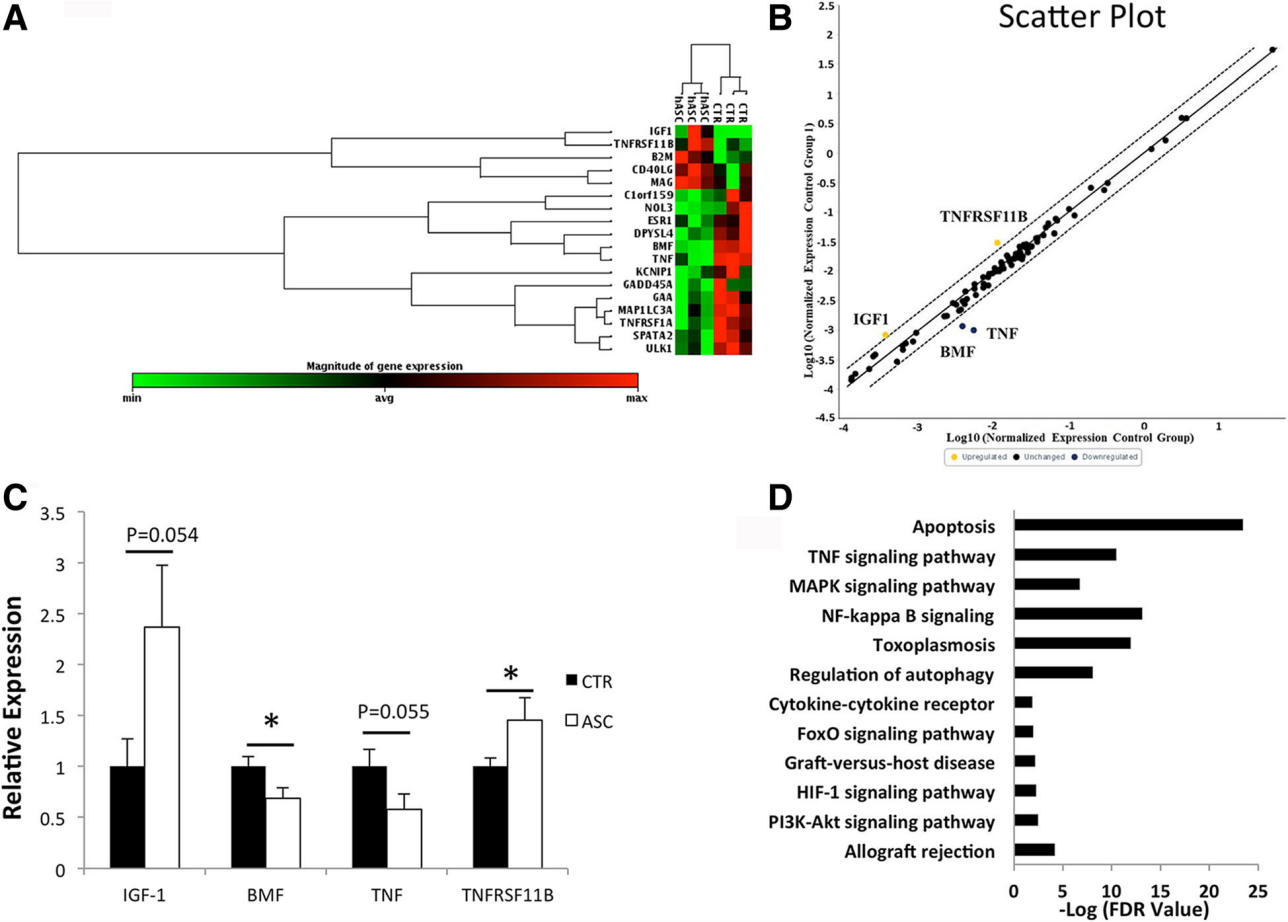
Transcriptional profile of apoptosis-related genes in human islet grafts transplanted alone or with CP-ASCs. **a** Heat map comparing gene expression (only selective genes exhibiting at least a 1.4-fold change or *p* < 0.05 from baseline are shown) in islets harvested on day 3 post transplantation from recipient mice that received islets alone or islets with CP-ASCs. Each *column* represents an islet graft from a single recipient. Gene expression shown by pseudocolor scale: *red*, upregulation of expression; *green*, downregulation of expression. **b** Scatter plot report of the results from the RT^2^ Profiler™ PCR Array experiment, indicating positions of several noteworthy genes based on the fold-changes in expression between the control group and the hASC group. *X* axis, normalized gene expression in control group; *Y* axis, normalized gene expression in hASC group. Boundary used was fold-change ≥ 2. **c** RT-PCR analysis of gene expression in control islets or CP-ASC cotransplanted islets.*p < 0.05, ﻿Student's t test. **d** GSEA performed to identify the enrichment pathway by *p* ≤ 0.05 and False discovery rate ( FDR) ≤ 0.05 using GSEA v2.2.1 software. Each *bar* represents an enriched pathway with significance determined using the –log FDR value (shown on *X* axis). *ASC* adipose-derived mesenchymal stem cell, *CTR* mice receiving islets alone, *BMF* Bcl-2 modifying factor, *IGF-1* insulin-like growth factor-1, *TNF* tumor necrosis factor, *TNFRSF11B* TNF Receptor Superfamily Member 11b (Color figure online)

Next, we assessed whether cotransplantation with CP-ASCs would suppress early islet death in human islets. Islets from nondiabetic cadaveric donors were transplanted under the kidney capsule of diabetic NOD-SCID mice. Transcriptional profiles of 84 apoptosis-related genes in islet grafts were measured by RT-PCR array 3 days post transplantation. Among the most changed genes, 10 genes were downregulated and 4 genes were upregulated at least 1.4-fold, the changes of 10 genes reached a significance (p<0.05), in ASC islets grafts compared to control islets (Fig. 4a). Among genes differentially expressed in ASC islets, TNF-α and BMF were downregulated, and IGF-1 and TNFSF11B were upregulated by at least 2-fold (Fig. 4b). The differences were confirmed by real-time RT-PCR analysis (Fig. 4c). Gene set enrichment analysis (GSEA) showed that TNF, NF-κB, and MAPK signaling pathways were suppressed, but pathways such as regulation of autophagy, cytokine–cytokine interaction, PI-3 K pathways, and others were upregulated (Fig. 4d). These results suggest a possible molecular basis for the protective effects of ASCs.

### ASCs secreted more IGF-1 when cocultured with islets

**Fig. 5:**
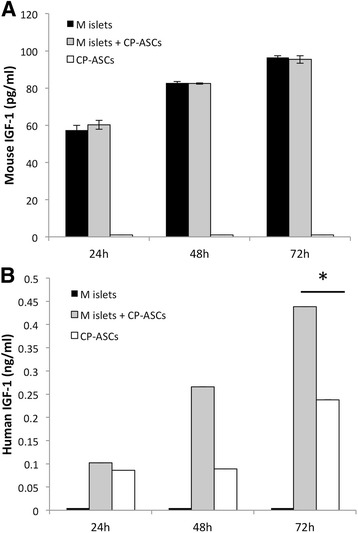
Secretion of IGF-1 in mouse islets cocultured with CP-ASCs. **a** Concentration of mouse IGF-1 in culture medium from mouse islets, mouse islets cocultured with CP-ASCs, and CP-ASCs alone after 24, 48, or 72 hours of culture. **b** Concentration of human IGF-1 in culture medium from mouse islets, mouse islets cocultured with CP-ASCs, and CP-ASCs alone after 24, 48, or 72 hours of culture. Data are representative of at least three individual experiments. *p < 0.05, Student’s *t* test. *M* mouse, *CP-ASC* adipose-derived mesenchymal stem cell from chronic pancreatitis patient, *IGF-1* insulin-like growth factor-1

We hypothesized that paracrine secretion of IGF-1 by CP-ASCs contributed to islet survival. To distinguish whether increased IGF-1 was from CP-ASCs or islets, we cocultured CP-ASCs and mouse islets in vitro, and measured the amounts of human IGF-1 (derived from CP-ASCs) and mouse IGF-1 (derived from islets) in the supernatants using ELISA kits for mouse or human IGF-1. At 24, 48, and 72 hours after initiation of CP-ASC and islet cocultures, no differences were observed in secreted mouse IGF-1 between mouse islets culture alone or with CP-ASCs (Fig. 5a). In contrast, secretion of human IGF-1 was significantly increased in CP-ASCs, and secretions of IGF-1 were even higher when CP-ASCs were cocultured with mouse islets for 48 and 72 hours (Fig. 5b), suggesting that IGF-1 was secreted by the CP-ASCs rather than by the islets, and that coculturing islets with CP-ASCs stimulated CP-ASCs to secrete more IGF-1 (Fig. 5b).

### Paracrine secretion of IGF-1 from CP-ASCs mediates the anti-apoptotic effects of CP-ASCs

**Fig. 6:**
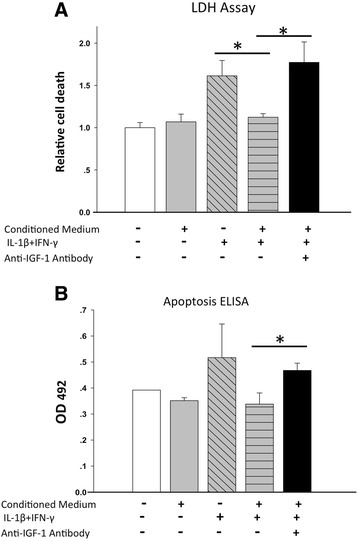
IGF-1 mediates the protective effects of ASC when challenged by proinflammatory cytokines. **a** Cell death measured by LDH assay in islets cultured in normal medium or in CP-ASC-conditioned medium and stimulated with proinflammatory cytokines in the presence or absence of anti-IGF-1 antibody. **b** Cell death measured by the Apoptosis ELISA kit in the same culture supernatants as **a**. Data are from at least three individual experiments. *p < 0.05 ANOVA with Bonferroni adjusted Student’s *t* test. *ELISA* enzyme-linked immunosorbent assay, *IGF-1* insulin-like growth factor-1, *IFN* interferon, *IL* interleukin, *LDH* lactate dehydrogenase

To determine whether IGF-1 secreted by CP-ASCs mediated the anti-apoptotic effects of CP-ASCs to islets, we cultured mouse islets in normal medium, conditioned medium, or conditioned medium containing the anti-IGF-1 antibody, and then challenged them with cytokines (IL-1β and IFN-γ). Islet apoptosis was measured and compared. Cytokine treatment induced significant apoptosis in islets as manifested by the generation of LDH (Fig. 6a). Islets cultured in conditioned medium from CP-ASCs showed significantly reduced cell death. Blocking human IGF-1 with the anti-IGF-1 antibody diminished the protective effects of CP-ASC-conditioned medium, suggesting that IGF-1 secreted by CP-ASCs is required for the anti-apoptotic effects of CP-ASCs. Similar results were obtained when apoptosis was measured using the Apoptosis ELISA kit (Fig. 6b). These data suggest that the anti-apoptotic effects of CP-ASCs were at least in part mediated by their paracrine secretion of IGF-1.

## Discussion

This study sheds light on the feasibility of cotransplanting autologous ASCs with islets for treatment of chronic pancreatitis. We found that cotransplantation of mouse islets with CP-ASCs into STZ-treated diabetic C57BL/6 mice led to better survival and function of transplanted islets, which was associated with inhibition of macrophage infiltration, reduced islet cell death, and improved angiogenesis post transplantation. Cotransplantation of CP-ASCs and islets in a NOD-SCID mouse model resulted in enhanced expression of IGF-1 and anti-apoptotic genes, including TNFRSF11B. This also suppressed expression of proinflammatory cytokines (TNF-α) and BMF in transplanted islets. In vitro, using an IGF-1 neutralizing antibody, we found that paracrine secretion of IGF-1 from CP-ASCs was required for their anti-apoptotic effects.

Cotransplantation of MSCs from various tissues improves islet graft survival in rodent models [20, 31, 32], and MSCs are currently being used in clinical trials for the treatment of autoimmune, inflammatory, and other diseases (www.clinicaltrials.gov). In most of these studies, allogeneic MSCs were used, which may be associated with safety and regulatory issues. In comparison, autologous MSCs are considered “minimally manipulated” biological products that require less stringent regulation by the FDA, and represent an attractive option for cell therapy. However, autologous MSCs are not always available for cell therapy because certain disease-related cellular, molecular, and biochemical changes mitigate the protective effects of autologous MSCs. For example, MSCs from patients with rheumatoid arthritis [33], immune thrombocytopenic purpura [34], and multiple sclerosis [35] showed defects in critical cell functions and capability in tissue repair. In contrast, MSCs from systemic sclerosis and patients with type 1 diabetes seem to retain their immunosuppressive and tissue protective capabilities [33, 36]. We have compared the cell phenotype, colony formation ability, multilineage differentiation ability, secretory characters, and immunosuppressive functions between MSCs harvested from healthy donors and CP patients. Our data suggested that CP-MSCs are similar to healthy MSCs, indicating that autologous MSCs may be suitable for cell therapy in CP patients undergoing TP-IAT. Here, we found that CP-ASCs retained characteristic and tissue protective properties when cotransplanted with islets. ASCs protected both mouse and human islets from inflammatory cytokine-induced death in islet cultures and islet grafts. The results of this study provide evidence that it will likely be beneficial to cotransplant autologous ASCs in patients undergoing autologous islet transplantation for treatment of chronic pancreatitis.

MSCs from different sources secrete different paracrine factors, and contribute to different aspects of protection in disease models [37]. For example, adipose-derived ASCs exhibited higher expression of IGF-1, VEGF-D, and IL-8, while MSCs from other sources including bone marrow, dermal tissue, and dermal papilla exhibited higher expression of VEGF-A, angiogenin, basic fibroblast growth factor (bFGF), and nerve growth factor (NGF) [38]. In an islet–MSC cotransplantation model, MSCs were shown to protect islets from cytokine-induced apoptosis mainly via their paracrine secretions of IL-6, VEGF-A, HGF, and TGF-β [39]. In our study, we found that CP-ASCs protected islets from cytokine-induced cell death via paracrine secretion of IGF-1, and through suppression of proinflammatory cytokines and death factors. Our data support the notion that CP-ASCs may be favored over other MSCs for augmenting therapeutic options for certain diseases.

In our study, we have infused patient-derived MSCs into a mouse model. It is noteworthy that MSCs have low immunogenicity because they lack expression of MHC class II and costimulatory molecules such as CD40, CD80, or CD86 [40]. The protective functions of human MSCs can therefore be evaluated in mouse models [41, 42].

IGF-1 mediates the therapeutic effects of ASCs in Duchenne muscular dystrophy and dystrophic muscle diseases [43]. IGF-1 also plays critical roles in islet/β-cell growth, survival, and metabolism under conditions of autoimmune-mediated or STZ-induced β-cell death [44]. Because our results also indicate that IGF-1 secreted by CP-ASCs protected islets from cell death, we suggest that IGF-1 treatment has promise to enhance survival of grafted islet cells [45, 46]. A recent study demonstrated that brown fat transplantation reduces hyperglycemia in type 1 diabetes in mice, primarily through an increase in circulating IGF-1 [47] and independent of insulin [48]. Therefore, we do not exclude the possibility that the ASC-released IGF-1 directly works on glucose homeostasis in our islet transplantation model. We also observed a dramatic increase in the expression of the anti-apoptotic gene, TNFRSF11B, in islet grafts cotransplanted with ASCs. Therefore, there is a possible role of other genes, such as TNFRSF11B, that might mediate the protective effects of ASCs.

The protective effects of ASCs were lasting. In the first few days after transplantation, ASCs suppressed macrophage infiltration and production of proinflammatory cytokines, and prevented early cytokine-induced islet death, possibly by the release of IGF-1. In the mean time, ASCs promoted angiogenesis, inhibiting potential islet cell death that might be caused by lack of vascularization. Eventually, without any other treatment, cotransplantation with ASCs resulted in the return of all recipient mice to normoglycemia.

In our transplantation model, islets were first cocultured with CP-ASCs, and then cotransplanted with CP-ASCs. Whehter coculture with CP-ASCs alone, or cotransplant with CP-ASCs alone and whether infusion of ASCs via other routes such as via intravenous injection can protect islets from death after transplantation are under investigation in our laboratory.

## Conclusion

Cotransplantation of islets with ASCs from chronic pancreatitis patients improved islet survival and function after transplantation. The effects appear mediated by their paracrine secretion of IGF-1, suppression of inflammation, and promotion of angiogenesis. ASCs from chronic pancreatitis patients have the potential to be used as a synergistic therapy to enhance the efficacy of islet transplantation.

## Abbreviations

ASC: Adipose-derived mesenchymal stem cell
BMF: Bcl-2 modifying factor
CP-ASC: Adipose-derived mesenchymal stem cell from chronic pancreatitis patient
HGF: Hepatocyte growth factor
IBMIR: Instant blood-mediated inflammatory reaction
IFN-γ: Interferon gamma
IL: Interleukin
IVGTT: Intravenous glucose tolerance test
MSC: Mesenchymal stem cell
TGF-β: Transforming growth factor beta
TNF: Tumor necrosis factor
TNFRSF11B: TNF Receptor Superfamily Member 11b
TP-IAT: Total pancreatectomy with islet autotransplantation
VEGF: Vascular endothelial growth factor
